# Incline and decline running alters joint moment contributions but not peak support moments in individuals with an anterior cruciate ligament reconstruction and controls

**DOI:** 10.3389/fspor.2023.1217783

**Published:** 2023-11-17

**Authors:** Kenneth Harrison, Hillary H. Holmes, Eric B. Finley, Keven Santamaria Guzman, Katherine C. Kimbrough, Jaimie A. Roper

**Affiliations:** School of Kinesiology, Auburn University, Auburn, AL, United States

**Keywords:** environmental demands, adaptation, anterior cruciate ligament, biomechanics, osteoarthritis, rehabilitation

## Abstract

Individuals with an anterior cruciate ligament reconstruction (ACLR) commonly exhibit altered gait patterns, potentially contributing to an increased risk of osteoarthritis (OA). Joint moment contributions (JMCs) and support moments during incline and decline running are unknown in healthy young adults and individuals with an ACLR. Understanding these conditional joint-level changes could explain the increased incidence of OA that develops in the long term. Therefore, this knowledge may provide insight into the rehabilitation and prevention of OA development. We aimed to identify the interlimb and between-group differences in peak support moments and subsequent peak ankle, knee, and hip JMCs between individuals with an ACLR and matched controls during different sloped running conditions. A total of 17 individuals with unilateral ACLR and 17 healthy individuals who were matched based on sex, height, and mass participated in this study. The participants ran on an instrumented treadmill at an incline of 4°, decline of 4°, incline of 10°, and decline of 10°. The last 10 strides of each condition were used to compare the whole-stance phase support moments and JMCs between limbs, ACLR, and control groups and across conditions. No differences in JMCs were identified between limbs or between the ACLR and healthy control groups across all conditions. Support moments did not change among the different sloped conditions, but JMCs significantly changed. Specifically, ankle and knee JMCs decreased and increased by 30% and 33% from an incline of 10° to a decline of 10° running. Here, the lower extremities can redistribute mechanics across the ankle, knee, and hip while maintaining consistent support moments during incline and decline running. Our data provide evidence that those with an ACLR do not exhibit significant alterations in joint contributions while running on sloped conditions compared to the matched controls. Our findings inform future research interested in understanding the relationship between sloped running mechanics and the incidence of deleterious acute or chronic problems in people with an ACLR.

## Introduction

Anterior cruciate ligament (ACL) tears are among the most prevalent musculoskeletal injuries, with an incidence of 68.6 in every 100,000 musculoskeletal injuries and an estimated $7 billion healthcare burden in the United States per year (1, 2). In addition to the prevalence of ACL tears and the financial burden of reconstruction and therapy, individuals with an ACL reconstruction (ACLR) can experience an upward threefold increased risk of developing osteoarthritis (OA) in the reconstructed knee (3–6). The prevalence of knee OA in the general population is around 13%, but over 50% of individuals with ACLR have been diagnosed with OA via radiographic evidence, as early as 12 years post-reconstruction (6–8). Because ACL tears commonly occur in adolescents and young adults, the onset and rate of OA development may occur dramatically earlier than in those without ACLR (5, 9, 10). Therefore, understanding biomechanical factors in individuals with an ACLR may mediate changes that can eventually lead to OA (11).

Both the ACL surgical repair and cartilage health post-ACLR surgery have been associated with altered knee mechanics during walking and running gait (3, 12–21). For example, individuals post-ACLR are reported to adopt a loading pattern with decreased external knee flexor moment in the reconstructed, or involved, limb (3, 15, 16, 21). This altered loading pattern may negatively contribute to knee cartilage health and OA development (14, 20). However, most literature on lower extremity mechanics in individuals with an ACLR is conducted on flat surfaces. Incline and decline surfaces are also found to alter joint mechanics and may have unique implications for individuals with an ACLR (22–26).

Environmental demands such as sloped surfaces may influence joint moment redistribution, which can be described through the concept of support moments and joint moment contributions (JMCs) (21, 25–28). Studying changes in JMCs can provide important insights into how individual joint mechanics adapt to achieve a particular support moment during gait (27, 28). Understanding how young adults with an ACLR modulate their JMCs compared to healthy controls during sloped treadmill running may provide additional rehabilitation implications on what training modalities to utilize or avoid (29). For example, in individuals 6 months post-ACLR, similar walking support moments were observed compared to healthy controls, but the support moment of the ACLR group consisted of the smaller knee and larger hip angular impulse contributions (21). Notably, the ankle, knee, and hip moments could redistribute their contributions in response to an ACLR but still produce a similar support moment (21, 27, 28).

In addition, kinematic changes to running patterns, such as decreases in step length, can lower peak compression forces in the knee of people with ACLR (30). These conditional changes in joint coordination could be vital to understanding subsequent pathology. If injured limbs adapt to the incline/decline conditions differently, it is necessary to measure what characteristics change and whether those are patterns that have previously been associated with OA development. If there was an association uncovered between knee JMC during sloped conditions and group (ACLR vs. CTRL), it could inform clinicians and coaches on what types of training modalities to avoid or limit the use of at specific points of the rehab timeline.

We aimed to investigate peak support moments and JMCs in individuals with an ACLR and healthy controls during running on different treadmill slopes to further understand pathological mechanics that could lead to OA development. In line with previous literature looking at the underloading of the affected limb (30), we hypothesized that individuals with ACLR would exhibit decreased knee JMCs in the involved limb compared to the uninvolved limb and decreased knee JMCs in the involved limb compared to the matched controls. Finally, we expected all the participants to display increased knee JMCs during decline running compared to incline running.

## Methods

### Participants

A total of 34 individuals, 17 with an ACLR (11 females and six males, age 21 ± 2 years, height 1.72 ± 0.08 m, mass 73.08 ± 11.17 kg) and 17 controls (11 females and six males, age 21 ± 3 years, height 1.74 ± 0.08 m, mass 71.65 ± 12.4 kg) participated in this case–control study (Table 1). The participants were recruited via flyers on campus, word of mouth, and SONA systems—an online extra credit platform used by the university. The inclusion criteria for the ACLR group required those previously sustaining an ACL, undergoing reconstructive surgery at least 6 months prior to participation in the study, and having medical clearance from a clinician for physical activity. All the participants answered “no” to questions on the National Academy of Sports Medicine Participation Activity Readiness Questionnaire (PAR-Q) to ensure safety in participation (31). The exclusion criteria for the ACLR group included those with less than 6 months post-ACL reconstruction and those who failed to gain medical clearance from a clinician. All individuals in the ACLR group had a unilateral tear with the sample containing 13 patellar tendon grafts, three hamstring grafts, and one quadriceps tendon graft. The control participants were matched to the ACLR participants based on stature (height and mass), followed by age, sex, and sports experience. All matches had an average difference in mass of less than 2 kg and an average difference in height of less than 5 cm. The inclusion criteria for the control group included those having no history of lower extremity or back injury requiring surgery or physical therapy and those being free of injury not requiring surgery for at least 1 year. Limb dominance was then determined for the control group by asking the participants which leg they would kick a soccer ball farthest with to match the demographics of the ACLR group (32). Before participation, all the participants reported that they were comfortable running incline and decline for at least 10 min in 1–3-min increments. The Institutional Review Board of Auburn University approved the investigation (Protocol #: 18-277 EP 1808), and study procedures were explained before the participants voluntarily signed informed consent.

**Table 1:** All participant characteristics.

	ACLR		CTRL		*p*	Cohen's *d*
Sex (F/M)	11F/7M		11F/7M		0.500	0.39
Age (years)	21 ± 2	19–26	22 ± 3	19–29	0.324	
Height (m)	1.72 ± 0.09	1.56–1.92	1.75 ± 0.08	1.635–1.91	0.891	0.35
Mass (kg)	73.10 ± 11.91	55.45–97.07	72.96 ± 11.81	54.43–97.98	0.972	0.012
Time post-reconstruction (months)	42 ± 23	6–77	—	—	—	—
KOOS subscore: pain	91.2 ± 6.8	75–100	96.3 ± 4.8	86–100	**0** **.** **018**	0.87
KOOS subscore: ADL	97.4 ± 3.9	46–100	99 ± 2.9	71–100	0.171	0.47
KOOS subscore: S and R	82.1 ± 15.3	88–100	96.5 ± 7.5	88–100	**0** **.** **001**	0.88
KOOS subscore: QoL	75.2 ± 5.2	50–100	95.3 ± 9.9	70–100	**<0** **.** **001**	2.54
KOOS subscore: symptoms	80.8 ± 11.9	44–100	94.6 ± 8.0	63–100	**0** **.** **001**	1.36

*p*-values in bold indicate statistically significant differences between groups. Values indicate mean ± SD. Values indicate range: minimum–maximum. M, male; F, female.

KOOS subscores: activities of daily living (ADL), quality of life (QoL), sports and recreation (S and R).

### Surveys and data collection

In a single laboratory visit, the participants completed the questionnaires and the running protocol. The ACLR and control groups completed the Knee Injury and Osteoarthritis Outcome Score (KOOS), a validated survey to assess knee function in individuals with an ACLR (33). KOOS subscores assessed pain, symptoms, activities of daily living, quality of life, and sports and recreation (33). The ACLR group reported information regarding time post-surgery and the level of activity before and after reconstruction through a questionnaire that we developed. Specifically, the question asked was “What do you do for exercise, and how many days a week, and for how long?”.

A 17-camera motion capture system (VICON, Vicon Motion Systems Ltd., Oxford, UK) collected kinematic data at 100 Hz, and a force-plate instrumented split-belt treadmill collected ground reaction forces at 1,000 Hz (Bertec Co., Columbus, OH, USA). Between each running trial, the participants stepped off the treadmill allowing the researcher to reset the force plates to zero in both the hardware and software to minimize noise artifacts.

### Testing protocol

**Figure 1:**
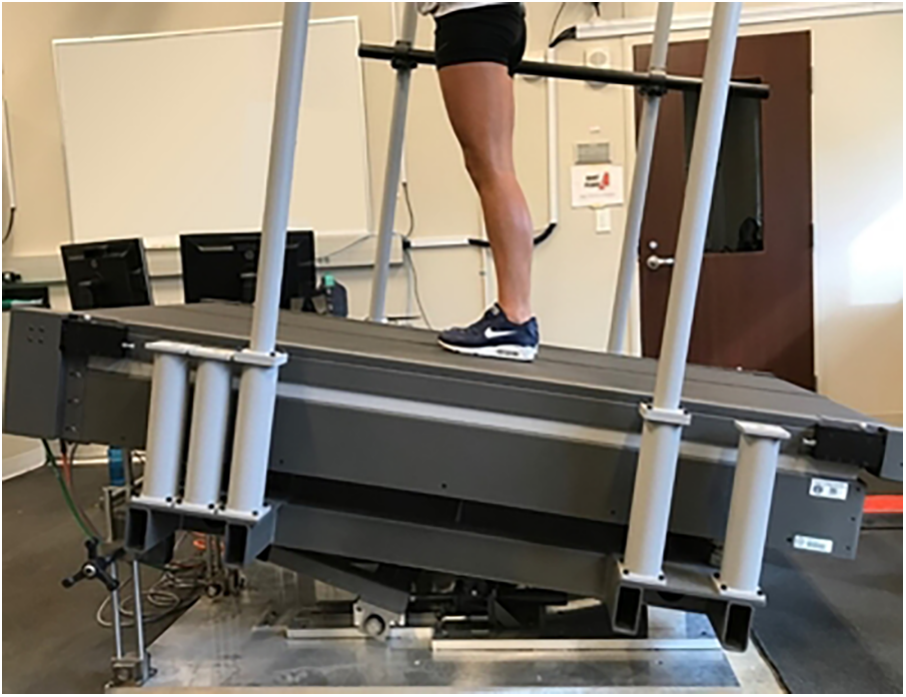
Instrumented treadmill used by participants shown at a 4 degree slope.

Each participant wore his or her own recreational gym attire including a t-shirt, shorts, and athletic shoes. The participants were then prepped with 26 reflective markers placed in accordance with the Vicon Plug-in Gait lower body functional AI marker set (VICON, Vicon Motion Systems Ltd., Oxford, UK). The participants were familiarized with the treadmill by running with no slope (flat) for 3 min at 2.5 m/s. The participants then ran on the treadmill for 1 min at a speed of 1.8 m/s under each of the following conditions: incline 4°, decline 4°, incline 10°, and decline 10° in that respective order (see Figure 1). Since running speed was reported to influence the magnitude of support moments more than the individual JMCs, it was important to implement a constant speed that was attainable across all slopes (34). A 2-min rest period was administered between all trials, and consistent instruction of when the treadmill was started and stopped was provided (35). Incline and decline angles were set in accordance with the prior literature (23, 24, 26). The trials were not randomized to mitigate the effect of fatigue on the participant. Running speeds were set slower than prior studies to prevent fatigue during incline running, given the increased metabolic cost for a given speed, and to mitigate potential joint discomfort during decline running, given decline running can increase patellofemoral stress (22–24, 26, 36, 37). During pilot testing, it was also determined that running any faster than 1.8 m/s at a 10° decline on a treadmill was very distressing and fear-inducing for the participants, inhibiting regular downhill running patterns. Flight phases were confirmed for all recorded trials and subsequent visual inspection of the data confirmed unimodal force curves that matched what is typically produced during running.

### Data analysis

Joint moments were calculated through the Vicon Nexus Plug-in Gait model (VICON, Vicon Motion Systems Ltd., Oxford, UK). All joint moments are reported as external moments in the sagittal plane. Kinematic and kinetic data were filtered at 6 and 50 Hz, respectively, using a fourth-order low-pass Butterworth filter. There is a lot of literature on the benefit of using the same filter frequency for kinematic and kinetic measures for high-impact movements (38–40) and in running (41). To confirm this effect did not skew our results, we tested our data with the same filter frequency and found no major effects for key outcome metrics or interpretations (42). Maximum hip flexion, knee flexion, and ankle dorsiflexion moments during the stance phase were recorded in both limbs and processed using a custom MATLAB code (2018a, MathWorks, Natick, MA, USA). Peak moments during the stance phase were normalized to body mass and averaged for the last 10 strides in the involved and uninvolved limbs. The involved limb of the control group was matched by limb dominance of the reconstructed limb of the ACLR group (i.e., if the involved ACLR limb was dominant, the dominant limb of the control was considered the involved limb). Whole-stance phase support moments were calculated in each limb as the sum of the averaged peak flexion joint moments of the last 10 strides during stance. JMCs were expressed as a percentage, calculated as the ratio of the averaged peak moments in the last 10 strides at one joint to the support moment of that limb (Equations 1 and 2) [adapted from (27, 28)]. The original calculation of JMCs is selected at the time point in which the support moment peak occurs, regardless of where the peak ankle, peak knee, or peak hip joint moment occurs. Because the ankle, knee, and hip peak joint moments likely occur at different time points, our calculation accounts for this. For example, the knee JMC is calculated at the time point where the peak knee joint moment occurs, instead of where the peak of the support moment occurs. JMCs at the hip, knee, and ankle were determined for the involved and uninvolved limbs for each running condition (see Supplementary Figure S1).

(1) SupportMoment=∑(PeakHip,Knee,AnkleJointMoment)

(2) JMC=[(PeakJointMoment)/(SupportMoment)]×100

### Statistical analysis

Testing was performed in SPSS 24 (IBM, Armonk, NY, USA). Independent *t*-tests compared participant characteristics (sex, height, mass, and age) between the ACLR and control groups. A 2 × 2 × 4 (group × limb × slope) mixed-model MANOVA determined the differences in support moments and JMCs among running slopes and between groups and between limbs. In the ACLR group, a 2 × 4 (limb × slope) within-subject MANCOVA determined if the results in the ACLR group limbs differed when covarying for the number of months post-reconstruction surgery. A Bonferroni correction was used during post-hoc testing to determine differences among conditions (slope) in both analyses. Any violation of Mauchly's test of sphericity was addressed using a Greenhouse–Geisser correction. A power analysis using G*Power version 3.1.9.7 (43) with preliminary data looking at joint moments in 10 young adults (five with ACLR and five matched controls) indicated a total sample size of 34 participants for significance using the MANOVA: special effects and interaction statistical test (1-*β* = 0.80, *α* = 0.05, effect size = 0.25).

## Results

### Demographics

The average time spent in physical therapy post-surgery was 5 ± 2 months. The average KOOS scores for the ACLR group were 91 for pain, 81 for symptoms, 97 for activities of daily living, 82 for sports and recreation, and 75 for quality of life. In total, 88% of our ACLR sample reported currently participating at the same pre-reconstruction levels of physical activity based on their responses to our developed questionnaire.

### Peak support moment and JMCs

**Figure 2:**
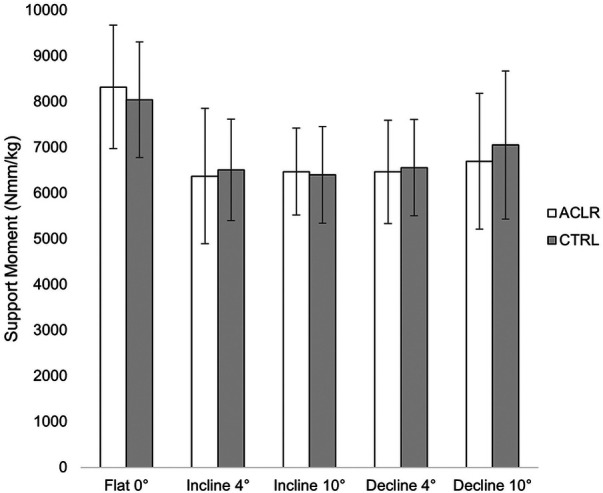
Total Support Moment between ACLR and CTRL groups.

There was no main effect of limb [Wilk's *ʎ* = 0.899, *F*(4.29) = 0.810, *η*^2^ = 0.101, *p* = 0.529] or group [Wilk's *ʎ* = 0.948, *F*(4.29) = 0.396, *η*^2^ = 0.052, *p* = 0.810, Table 2]. However, there was a main effect of the condition [Wilk's *ʎ* = 0.025, *F*(12,246.346) = 45.827, *η*^2 ^= 0.645, *p* < 0.001]. Univariate tests indicated significant differences among conditions in ankle JMCs [*F*(1.828, 58.496) = 227.149, *η*^2 ^= 0.877, *p *< 0.001], knee JMCs [*F*(1.819, 658.199) = 405.025, *η*^2 ^= 0.927, *p* < 0.001], and hip JMCs [*F*(1.920,61.443) = 5.428, *η*^2 ^= 0.145, *p* = 0.004], but not support moments [*F*(2.269, 72.600) = 2.972, *η*^2 ^= 0.085, *p *= 0.051, Figure 2]. Due to sphericity being violated, Greenhouse–Geisser corrected values are reported for the main effects of condition (*ε* = 0.756) and univariate tests on JMC of the ankle (*ε* = 0.560), knee (*ε* = 0.607), and hip (*ε* = 0.526). No significant interaction effects were found for support moments or JMCs.

**Table 2:** Main effect of limb and main effect of group in support moment and JMCs.

Measure	Limb	Group	*M*	*SE*	95% confidence interval	
					Lower bound	Upper bound
Support moment (N m kg^−1^)	Involved	CTRL	7.0	0.3	6.4	7.5
		ACLR	6.8	0.3	6.2	7.3
	Uninvolved	CTRL	6.8	0.3	6.3	7.4
		ACLR	7.0	0.3	6.4	7.5
Ankle JMC (%)	Involved	CTRL	39.2	1.6	35.9	42.5
		ACLR	41.3	1.6	38.0	44.6
	Uninvolved	CTRL	40.4	1.4	37.4	43.3
		ACLR	40.3	1.4	37.3	43.2
Knee JMC (%)	Involved	CTRL	30.0	1.5	27.0	33.0
		ACLR	28.7	1.5	25.7	31.7
	Uninvolved	CTRL	29.2	1.3	26.5	31.9
		ACLR	30.3	1.3	27.6	33.0
Hip JMC (%)	Involved	CTRL	30.8	2.1	26.4	35.1
		ACLR	30.0	2.1	25.6	34.4
	Uninvolved	CTRL	30.5	2.0	26.5	34.5
		ACR	29.4	2.0	25.4	33.4

**Figure 3:**
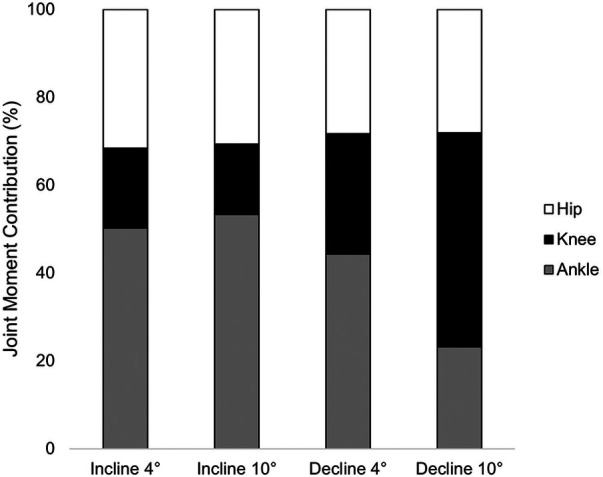
Averaged JMCs across conditions in all participants.

Ankle JMCs did not differ between incline 4° and incline 10° running (*p* = 0.569). Compared to those during incline 4° running, ankle JMCs were 22% and 27% smaller during decline 4° and decline 10° running, respectively (*p* < 0.001). Compared to those during incline 10° running, ankle JMCs were 25% and 30% smaller during decline 4° and decline 10° running, respectively (*p* < 0.001). Ankle JMCs were 5% smaller during decline 4° running compared to those during decline 10° running (*p* < 0.001). Knee JMCs between incline 4° and incline 10° were not significantly different (*p* = 0.387). However, knee JMCs during decline 4° running were 26% larger than those during incline 4° (*p* < 0.001) and 28% larger than those during incline 10° (*p* < 0.001). Knee JMCs during decline 10° were 31% larger than those during incline 4° (*p* < 0.001) and 33% larger than those during incline 10° (*p* < 0.001). Hip JMCs were 3% smaller during decline 4° running compared to those during incline 10° running (*p* = 0.035). No other significant differences in hip JMCs across conditions were present (*p* ≥ 0.05, Table 3, Figure 3). Covarying for the time post-reconstruction surgery did not affect the condition [Wilk's *ʎ* = 0.254, *F*(12,111.413) = 0.383, *η*^2^ = 0.035, *p* = 0.967] or limb [Wilk's *ʎ* = 0.629, *F*(12.4) = 1.773, *η*^2^ = 0.101, *p* = 0.289].

**Table 3:** Support moment and joint moment contributions by condition.

Measure	Slope	*M*	*SE*	95% confidence interval		Difference from incline 4°		Difference from incline 10°		Difference from decline 4°	
				Lower bound	Upper bound	*p*	*d*	*p*	*d*	*p*	*d*
Support moment (Nm/kg)	Incline 4°	6.4	6.4	0.2	6.0	—	—	—	—	—	—
	Incline 10°	6.4	6.4	0.2	6.1	1.000	0.003	—	—	—	—
	Decline 4°	6.5	6.5	0.2	6.1	1.000	0.063	1.000	0.076	—	—
	Decline 10°	6.9	6.9	0.3	6.3	0.311	0.315	0.259	0.348	0.206	0.280
Ankle JMC (%)	Incline 4°	50	2	46	54	—	—	—	—	—	—
	Incline 10°	53	1	50	56	0.569	0.326	—	—	—	—
	Decline 4°	28	1	27	30	**<0** **.** **001**	2.645	**<0** **.** **001**	3.751	—	—
	Decline 10°	23	1	21	25	**<0** **.** **001**	3.227	**<0** **.** **001**	4.447	**<0** **.** **001**	1.011
Knee JMC (%)	Incline 4°	18	1	17	20	—	—	—	—	—	—
	Incline 10°	16	1	13	19	1.000	0.387	—	—	—	—
	Decline 4°	44	1	42	47	0.490	4.922	**<0** **.** **001**	4.308	—	—
	Decline 10°	49	2	46	52	0.718	4.632	**<0** **.** **001**	4.279	**<0** **.** **001**	0.611
Hip JMC (%)	Incline 4°	32	2	27	36	—	—	—	—	—	—
	Incline 10°	31	1	29	33	1.000	0.098	—	—	—	—
	Decline 4°	27	1	25	30	0.429	0.461	**0** **.** **035**	0.461	—	—
	Decline 10°	28	2	24	32	0.431	0.308	0.559	0.290	0.431	0.071

*d* indicates Cohen's *d*, and bold values are significant *p* < 0.05.

## Discussion and implications

We analyzed peak support moments and JMCs to better understand mechanics during cyclical loading produced during incline and decline running in individuals with an ACLR. Our main findings were as follows: (1) individuals with ACLR exhibited no differences between limbs in JMCs; (2) individuals with ACLR did not display alterations in JMCs and peak support moments compared to those without ACLR; and (3) JMCs, particularly at the ankle and knee, changed between incline and decline running, but peak support moments did not.

We did not identify limb asymmetries or differences in the involved limb between the ACLR and CTRL groups in peak support moment or JMCs. The lack of differences may be due to factors contributing to knee function and physical activity levels. For example, 88% of our ACLR sample achieved pre-reconstruction physical activity levels. In one ACLR cohort, 92% of participants returned to fully competitive sports 8 months post-operation (44, 45). Our sample of people with ACLR may have been too far in time from the initial injury/surgery to have retained meaningful changes in joint coordination. Prior evidence suggests that there may be a relationship between time and changes to gait mechanics in people with ACLR (45–47). Because of this, we evaluated if the time post-reconstruction surgery influenced peak support moments and JMCs in the ACLR group among conditions and between limbs to control for participants who had more time since surgery to return to baseline mechanics. It is also possible that other factors, such as graft type, meniscal pathology, rehabilitation protocol, and sex, were stronger indicators of asymmetry and were not included in our analysis.

We observed changes in ankle and knee JMCs but not peak support moments between incline and decline running. Our results support the notion that lower extremity joints can adjust and redistribute to produce a constant propulsive moment in gait (26–28). For example, we observed an increase in knee JMC by 33% when running at an incline of 10° compared to a decline of 10°, which corroborates previously described common patterns in sloped running mechanics. Knee extensor impulse increased by 54% when running at a 10° incline compared to running at a 10° decline overground. Similarly, hip and ankle angular impulse increased by 177% and 46%, respectively (26). Differences in methods should be noted in comparing and interpreting results. For example, DeVita et al. (26) implemented a 5-m ramp between 6-m flat runways. In contrast, our investigation involved an instrumented treadmill set at speed for the entirety of the incline and decline conditions. Our investigation and DeVita et al. (26) reported constant velocities in the recorded trials; however, our investigation may display how individuals adapt mechanics to a longer, continuous duration of sloped running at a slower speed (26). Taken together with the results reported by DeVita et al. (26), the duration, speed, and environment (treadmill vs. overground) may be of importance to consider (26, 34). Our participants ran at a slower speed (1.8 m/s) in comparison to other studies ranging from 3.13 to 3.35 m/s (25, 26). We selected a set, slower speed to mitigate potential joint stress, the influence of speed on mechanics, and the effects of fatigue on mechanics.

Increases in knee loading during decline running could be due to the eccentric demand on the knee extensor musculature during decline locomotion (48). Importantly, abnormal joint motions that cause rapid shifts in loading after an ACLR are proposed to influence the metabolic response of cartilage, which may accelerate the onset of OA (3, 12). Decline running has been previously recognized as a return-to-play landmark due to increased difficulty (49). Recognizing this change in knee JMCs for incline and decline conditions could provide implications for rehabilitation and return-to-play protocols. Trainers and clinicians should be cognizant of the increased moment placed on the knee during slow decline running.

This investigation comes with limitations. First, only measuring peak joint moments does not consider how mechanics may differ through the entire stance phase. However, the calculation used here was revised to appreciate loading patterns at three time points of a gait cycle rather than the original JMC calculation, which only considers loading at one time point. We ran a secondary analysis looking at only peak joint moments (without reducing to support moments or JMC) and found the same interpretation and results. Second, compared to other investigations, our participants ran at slower speeds (22, 25, 26). However, rather than allowing participants to self-select a running speed, we selected this slower speed so we could understand the true contribution of the slope without obscuring our results with speed differences across the sloped conditions. In addition, we selected this speed to ensure that the participants could comfortably complete all incline and decline trials without inducing a large amount of fatigue. Third, we did not randomize the condition sequence of incline and decline running with varying slopes for each trial which could have caused an experimental order effect. All participants ran at an incline of 4°, decline of 4°, incline of 10°, and decline of 10° in this respective order. This specific order was chosen due to feedback on participant comfort during pilot testing and the time it takes to reconfigure the treadmill. Fourth, our investigation did not control for participant running experience level or shoe type. Athletic shoes were not provided for each participant; thus, each participant wore their personal athletic shoes. Differences in footwear have been shown to impact lower limb kinetics during walking and running (50, 51). However, we aimed to have differences in footwear during lab testing closely mimic the differences in footwear observed in the real world. Lastly, our investigation did not include direct measures of cartilaginous health or OA development, nor did we control for graft type in the analysis. Future investigations should consider additional variables such as radiographic measures of cartilaginous health, foot strike pattern, shoe type, running experience, and more comprehensive kinetic measures to provide more direct implications on the influence of sloped running on OA development.

## Conclusion

Individuals with an ACLR exhibit similar peak support moments and JMCs during incline and decline treadmill running compared to people without ACLR. Further, we did not observe asymmetries in JMCs or support moments between limbs. We observed that different slopes influence JMCs, particularly at the ankle and knee, without influencing the support moment. Our data provide evidence that those with ACLR do not exhibit significant alterations in joint contributions while running on sloped conditions compared to the matched controls. Our findings inform future research interested in understanding the relationship between sloped running mechanics and the incidence of deleterious acute or chronic problems in people with an ACLR.

Notably, the increases in knee JMCs during decline running may provide insight into joint health in individuals who underwent an ACLR and are at heightened risk for cartilage degeneration. A relevant clinical implication for our findings could be incorporating incline and decline overground or treadmill training within return to sports protocol following an ACLR. In addition, incline and decline locomotions are a part of the real world. Implementing a rehabilitation protocol in which periodic increases in the incline and decline angles during training may benefit individuals post-ACLR by promoting effective movement adjustments to decrease risk factors such as possible reinjury or OA.

## Data Availability

The datasets presented in this study can be found in online repositories. The names of the repository/repositories and accession number(s) can be found in the article/**Supplementary Material**.
