# Supplementary material for: Incline and decline running alters joint moment contributions but not peak support moments in individuals with an anterior cruciate ligament reconstruction and controls
Source: Front Sports Act Living. 2023 Nov 17;5:1217783. doi: 10.3389/fspor.2023.1217783 (PMC10691489; doi:10.3389/fspor.2023.1217783)
Supplement: Supplementary file 1 [file Datasheet1.pdf]

SUPPORT MOMENT CALCULATION METHODS TO REPRESENT PEAK FORCES DURING INCLINE/DECLINE RUNNING IN ACLR PATIENTS

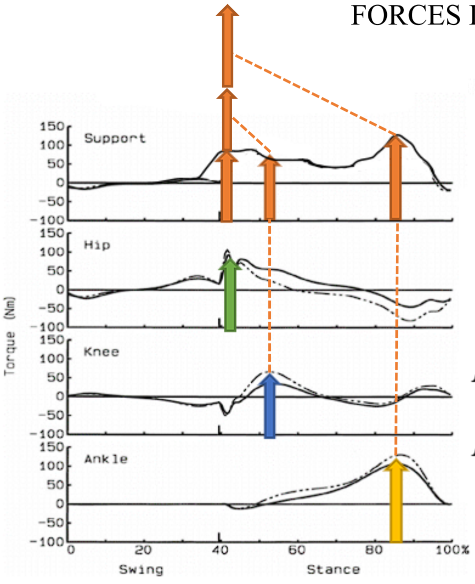

Method 1: Stance Phase JMC

Peak moment of each stance phase per joint was averaged over 5 strides

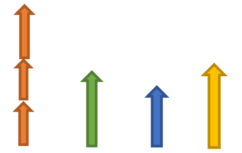

$$Peak\ Ms = Mh + Mk + Ma$$

$$Peak\ knee\ JMC\% = \frac{Mk}{Ms} \times 100$$

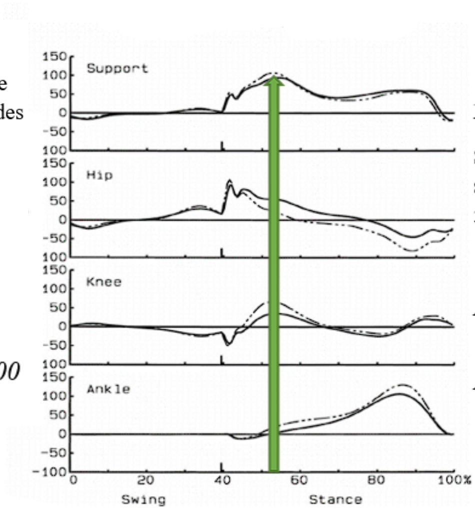

Method 2: Support Frame JMC

Stance frame of average peak support over 5 strides was used to isolate joint moment frames

$$Peak\ Ms = Mh + Mk + Ma$$

$$Peak\ knee\ JMC\% = \frac{Mk}{Ms} \times 100$$

Method 1 (left side): What we used in this current manuscript

For every stance phase, the peak moment of the hip (Mh), knee (Mk), and ankle (Ma) are summed together to calculate the whole-stance-phase support moment (Ms).

To find the JMC of the knee using this method, you would take that whole-stance-phase support moment (Ms) and use it as a denominator under the peak knee moment (Mk) in Winters equation.

This method informs us how the joints work together to generate peak forces required to remain upright throughout stance phase, rather than during a single moment in stance phase.

Method 2 (right side): What is traditionally done

For every stance phase, you isolate the frame where the support moment is at its peak. From this frame you pull the joint moments of the hip, knee and ankle and perform the regular JMC calculation.

This informs us about the role of each joint in achieving a single support moment for a specific moment in time. Rather than showing its role through stance phase, this tells us about their role during a specific component of stance phase (for example, heel strike).

Justification:

Since we are interested in investigating changes in forces and overall mechanics of the joint that can be associated with cartilage health, we decided Method 2 would potentially miss out on peak forces being experienced during points of stance phase not in line with the peak support moment frame.

We hope our composite measure can help build upon the traditional JMC calculation depending on the research questions being asked.
